# A municipality-level, monthly dataset of disappearance cases and outcomes in Mexico from the National Registry of Disappeared Persons, 2015–2025

**DOI:** 10.1016/j.dib.2026.112897

**Published:** 2026-05-29

**Authors:** Marco A. Escobar, Gerardo Reyes-Guzman, Abraham Sanchez Ruiz

**Affiliations:** a Facultad de Ingenierías y Tecnologías, Universidad La Salle Bajío, *Av*. Universidad 602, Lomas del Campestre, León, Guanajuato 37150, Mexico; b Facultad de Economía, Benemérita Universidad Autónoma de Puebla, *Av*. San Claudio s/n, Ciudad Universitaria, Puebla, Puebla 72570, Mexico; c Facultad de Estudios Superiores, Universidad La Salle Bajío, Libramiento a Morelia km 7.5, Poblado de San Juan de Razos, Salamanca, Guanajuato 36700, Mexico

**Keywords:** Missing persons, RNPDNO, Administrative records, Spatiotemporal data, Human rights, Open data, Mexico, Web scraping

## Abstract

>132,000 persons are officially registered as disappeared and not located in Mexico, yet the government registry that documents this crisis, the National Registry of Disappeared and Unlocated Persons (*Registro Nacional de Personas Desaparecidas y No Localizadas*, RNPDNO), provides no public API, no bulk-download facility, and no machine-readable data export. The portal employs session-based authentication and CAPTCHA verification; individual queries return dynamically rendered HTML tables for single municipality–status–date combinations. An original extraction pipeline (Python, requests, BeautifulSoup4) systematically queried the portal across all combinations of 2478 municipalities, four victim-status categories, and monthly date ranges from January 2015 through December 2025. Raw records were processed through a deterministic cleaning pipeline with two-pass geographic normalization against the INEGI municipal geo-code catalog. The dataset is organized into four CSV files by victim-status category: (i) all registered persons (status_id = 0; 57,373 rows); (ii) disappeared and not located (status_id = 7; 34,617 rows); (iii) located alive (status_id = 2; 37,379 rows); (iv) located deceased (status_id = 3; 9936 rows). Each row represents a unique municipality–year–month cell with sex-disaggregated counts. The AGEEML-normalized cvegeo enables direct spatial joins to INEGI cartographic and census data that the portal's free-text municipality strings preclude. Four sentinel geo-codes flag 4141 rows (12,210 persons) with unresolvable geographic information. The complete pipeline and all manual override files are released in an open-source repository.

Specifications Table

**Table:** 

Subject	Social Sciences
Specific subject area	Criminology; enforced disappearances; human rights data; administrative records
Type of data	Table, Figure Processed, Raw
Data collection	Data were acquired via automated web scraping (Python 3.10, requests, BeautifulSoup4) of the National Registry of Disappeared and Unlocated Persons (*Registro Nacional de Personas Desaparecidas y No Localizadas*, RNPDNO) sociodemographic portal. The scraper sent POST requests across 33 states, four victim-status categories (total, disappeared/not located, located alive, located deceased), and 132 monthly periods (January 2015 –December 2025); each response returned municipality-level counts for a single state–status–month combination. Geographic normalization used a two-pass join against the 2023 edition of the Single Catalogue of Geo-statistical Area Keys (*Catálogo Único de Claves de Áreas Geoestadísticas Estatales, Municipales y Localidades*, AGEEML), published by the National Institute of Statistics and Geography (*Instituto Nacional de Estadística y Geografía*, INEGI), supplemented by 25 state-level corrections, 36 manual overrides, and sentinel codes for unresolvable records. Sex-disaggregated counts (male, female, undefined) were extracted per municipality–month cell.
Data source location	Primary source Country: Mexico. Institution: National Search Commission (Comisión Nacional de Búsqueda, CNB). Source URL: https://versionpublicarnpdno.segob.gob.mx/Dashboard/Sociodemografico Geographic reference Institution: INEGI Catalog: AGEEML (2023). URL: https://www.inegi.org.mx/app/ageeml/
Data accessibility	Repository name: Open Science Framework (OSF) Data identification number: DOI 10.17605/OSF.IO/4AYMZ Direct URL to data: https://osf.io/4aymz/ Code repository: https://github.com/marco-escobar/rnpdno-scraper Instructions for accessing these data: The OSF repository contains four processed CSV files (rnpdno_total.csv, rnpdno_disappeared_not_located.csv, rnpdno_located_alive.csv, rnpdno_located_dead.csv) and four intermediate Parquet files. All files are publicly accessible without registration. The GitHub repository contains the complete extraction and processing pipeline, manual override files, and the Conda environment specification (environment.yml) required to reproduce the dataset.
Related research article	None

## 1 Value of the Data

• The four-file structure by victim-status category (total registered, disappeared and not located, located alive, located deceased) enables outcome-transition analyses, including location rates, resolution-time proxies, and spatial heterogeneity in case outcomes, that single-status or single-city extractions cannot support.
• The dataset enables research designs across four disciplinary communities. For criminology, the outcome-status panel supports spatial-econometric analyses of resolution-rate heterogeneity. For human-rights research, the cross-status structure permits empirical characterization of the documentation gap in the national registry. For public health, sex-disaggregated municipality–month counts support gender-stratified missing-population estimation. For public policy, monthly resolution across 132 periods enables event-study designs around the 2017 enactment of the General Law on the Forced Disappearance of Persons (*Ley General en Materia de Desaparición Forzada de Personas*, LGMDFP) and its subsequent reforms.
• Monthly temporal resolution across 132 periods (January 2015 – December 2025) supports sub-annual analyses: event-study designs around policy interventions, seasonal decomposition, and lagged-response modeling. These analyses are not possible with the annual or cumulative snapshots used in prior municipality-level work on disappearances in Mexico.
• Each record carries a standardized five-digit INEGI geo-code (cvegeo) assigned through two-pass normalization against the official AGEEML municipal catalog. This enables direct spatial joins to cartographic boundaries, population projections, and census microdata without intermediate reconciliation — a prerequisite for population-normalized rate computation and spatial cluster detection methods such as LISA and Getis-Ord Gi*.

## 2 Background

Large-scale disappearances in Mexico have been documented since the 1960s [1], but the phenomenon accelerated after 2006 [2] with the federal government's militarized strategy against drug trafficking organizations. The 2017 LGMDFP mandated a national registry, resulting in the RNPDNO, administered by the CNB [3,4]. As of early 2026, the registry contains >132,000 persons classified as disappeared and not located. In February 2026, the Inter-American Commission on Human Rights issued a thematic country report on disappearances in Mexico [5]; the following month, the United Nations (UN) Committee on Enforced Disappearances referred the situation in Mexico to the UN General Assembly under article 34 of the Convention for the first time in the Convention's history [6].

Prior quantitative work has relied on annual snapshots, single-city extractions, or aggregate national totals [[7], [8], [9]]. The RNPDNO portal provides no API, no bulk download, and no machine-readable export; queries require CAPTCHA verification and return dynamically rendered HTML for single municipality–status–date combinations. This dataset was compiled to provide a complete, municipality-level, monthly extraction of all four RNPDNO victim-status categories at national scale [10].

## 3 Data Description

The dataset is hosted on OSF [11] and organized into three directories. The processing pipeline and all supporting code are maintained in a separate GitHub repository [12].

### 3.1 Repository structure

osf-4aymz/

├—— processed/

│ ├—— rnpdno_total.csv

│ ├—— rnpdno_disappeared_not_located.csv

│ ├—— rnpdno_located_alive.csv

│ └—— rnpdno_located_dead.csv

├—— raw/

│ ├—— rnpdno_total_2015_2025.parquet

│ ├—— rnpdno_desaparecidas_no_localizadas_2015_2025.parquet

│ ├—— rnpdno_localizadas_con_vida_2015_2025.parquet

│ └—— rnpdno_localizadas_sin_vida_2015_2025.parquet

├—— docs/

│ └—— quality_report.csv

└—— README.md

Together with Table 1 (schema) and Table 2 (sample records), this directory tree constitutes the structural overview of the dataset.

**Table 1:** Schema of the four processed RNPDNO CSV files. All four files share this schema. Range columns report empirical min–max observed across the v1.0.0 release (*n* = 139,305 rows).

Column	Type	Range	Description
cvegeo	string	01,001–32,570; sentinels: 99,998, 99,999, XX998, XX999	Five-digit composite INEGI geo-code (cve_estado + cve_mun); zero-padded. Sentinel values: 99,998 (unknown state), 99,999 (unresolved state), XX998/XX999 (municipality unspecified/unknown).
cve_estado	string	``01''–``32''; ``99'' sentinel	Two-digit INEGI state code, zero-padded (01–32). Value 99 reserved for records with unresolved state assignment.
state	string	32 official names; ``Unresolved''	Official state name (Spanish), normalized from the INEGI catalog.
cve_mun	string	``001''–``570''; ``998'', ``999'' sentinels	Three-digit INEGI municipal code, zero-padded (001–570). Sentinel values: 998 (no municipal reference), 999 (municipality unknown).
municipality	string	Free text	Official municipal name (Spanish), normalized from the INEGI catalog.
male	integer	0–93	Count of male persons.
female	integer	0–98	Count of female persons.
undefined	integer	0–18	Count of persons with indeterminate or unreported sex.
total	integer	1–158	Total persons (male + female + undefined).
year	integer	2015–2025	Calendar year (2015–2025).
month	integer	1–12	Calendar month (1–12).
status_id	integer	{0,2,3,7}	RNPDNO victim-status code: 0 = total, 7 = disappeared and not located, 2 = located alive, 3 = located deceased.

**Table 2:** Sample rows illustrating regular and sentinel records.

Field	Regular row	Sentinel XX998	Sentinel 99,999
cvegeo	11,020	02,998	99,999
cve_estado	11	02	99
state	guanajuato	baja california	Unresolved
cve_mun	020	998	999
municipality	leon	sin municipio de referencia	pueblo nuevo
male	4	1	1
female	3	0	0
undefined	0	0	0
total	7	1	1
year	2024	2024	2025
month	6	4	5
status_id	7	7	7

### 3.2 Processed CSV files

All four processed files share the twelve-column schema described in Table 1. Each row represents one (municipality, year, month, status) observation. Geo-codes conform to the INEGI municipal geo-code catalog. Counts represent persons reported within the given municipality, month, and year. Of the 2478 municipalities in the AGEEML catalog, 1959 are observed at least once in the panel; the remaining 519 record no cases over the 2015–2025 window.

Table 2 illustrates the schema with three sample rows drawn from rnpdno_disappeared_not_located.csv: a regular municipality record, a sentinel record for a known state with no specified municipality, and a sentinel record for fully unresolved geography.

**rnpdno_total.csv** contains 57,373 rows corresponding to all persons registered in the RNPDNO, regardless of subsequent location status (status_id = 0). Temporal coverage spans January 2015 through December 2025 (up to 132 monthly periods per municipality).

**rnpdno_disappeared_not_located.csv** contains 34,617 rows for persons classified as disappeared and not yet located (status_id = 7). This file is the primary analytical target for spatiotemporal characterization of active disappearance caseloads.

**rnpdno_located_alive.csv** contains 37,379 rows for persons subsequently located alive (status_id = 2).

**rnpdno_located_dead.csv** contains 9936 rows for persons located deceased (status_id = 3).

**Fig. 1:**
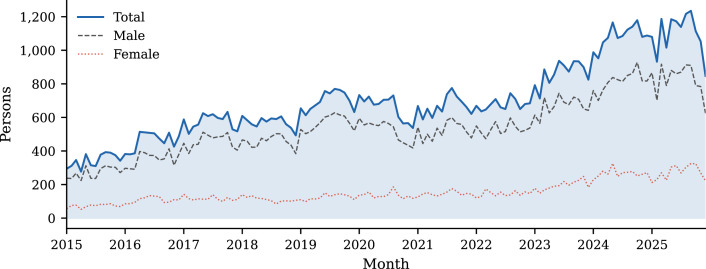
Monthly national count of persons disappeared and not located (status_id = 7), January 2015 – December 2025, disaggregated by sex. Records with unknown state are excluded.

**Fig. 2:**
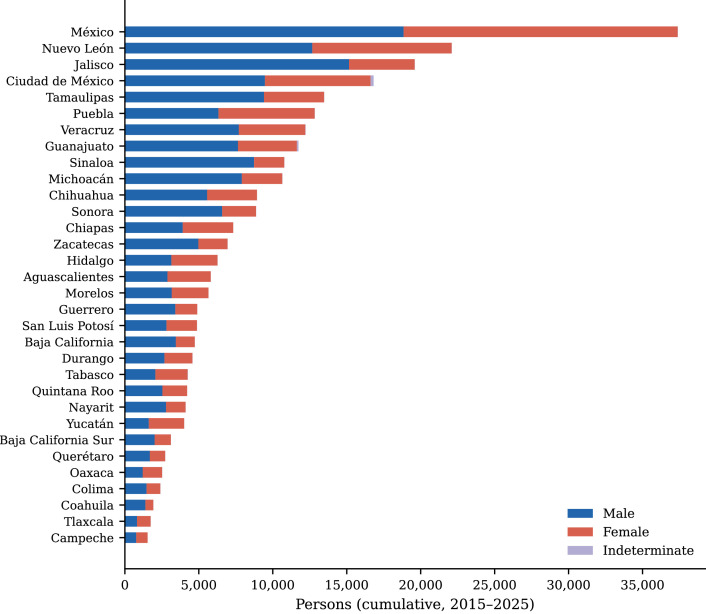
Cumulative registered persons by state for all statuses (status_id = 0), January 2015 – December 2025, ranked by total volume.

Of the 2478 municipalities in the AGEEML 2023 catalog, 1959 are observed at least once in the panel across the four files; the remaining 519 record no cases over the 2015–2025 window. The long-format files contain only municipality–month–status cells with at least one registered person; users requiring a balanced municipality × month × status panel should zero-infill at load time. Fig. 1 plots the national monthly count of the disappeared-and-not-located series (status_id = 7), disaggregated by sex, across the 132-month window; Fig. 2 ranks the 32 states by cumulative registered persons across all statuses (status_id = 0).

### 3.3 Sentinel geo-code records

A subset of rows in each file carries sentinel geo-codes indicating unresolvable geographic information in the source data (Table 3). These rows are retained rather than discarded to preserve the original aggregate counts. For municipality-level spatial analyses, the minimum filter is cvegeo % 1000 < 998, which excludes all four sentinel categories simultaneously while preserving every regular municipality. Aggregate analyses at the state level should retain XX998 and XX999 rows (where the state is known but the municipality is not) and may optionally retain 99,998 (unknown state) depending on whether unattributed cases should contribute to national totals. A given municipality–month cell may appear in multiple CSV files if cases with different outcomes were registered there.

**Table 3:** Sentinel geo-codes assigned to records with unresolvable geographic information, summed across all four processed CSV files.

cvegeo	Condition	Rows	Persons
99,998	Unknown state (id_estado = 33)	198	436
99,999	Unresolved state assignment	4	4
XX998	No municipal reference	1749	3978
XX999	Municipality unknown	2190	7792
**Total**		**4141**	**12,210**

### 3.4 Intermediate parquet files

Four intermediate Parquet files in raw/ contain the consolidated raw records after scraping but prior to geographic normalization. These files retain the original column names and free-text municipality strings from the RNPDNO portal and are provided for provenance purposes. Filenames reflect the original Spanish terminology used in the source system.

### 3.5 Quality report

The file docs/quality_report.csv contains per-dataset quality metrics generated at the end of each pipeline run, including row counts, sex–total consistency checks, and sentinel-record counts. As of the reference extraction (March 2026), all four processed files pass schema validation; zero sex–total inconsistencies are present; and exact duplicate rows originating from source-data artefacts have been removed.

### 3.6 Open data and reuse

The dataset is aligned with the FAIR Guiding Principles [14] and released under a CC-BY 4.0 license, following established practice for openly released compiled public-sector datasets [15]. A minimal reproducible quick-start (data loading and a worked example) is maintained in the GitHub repository [12] and the OSF deposit [11] rather than reproduced here, to keep the article focused on dataset structure.

## 4 Experimental Design, Materials and Methods

**Fig. 3:**
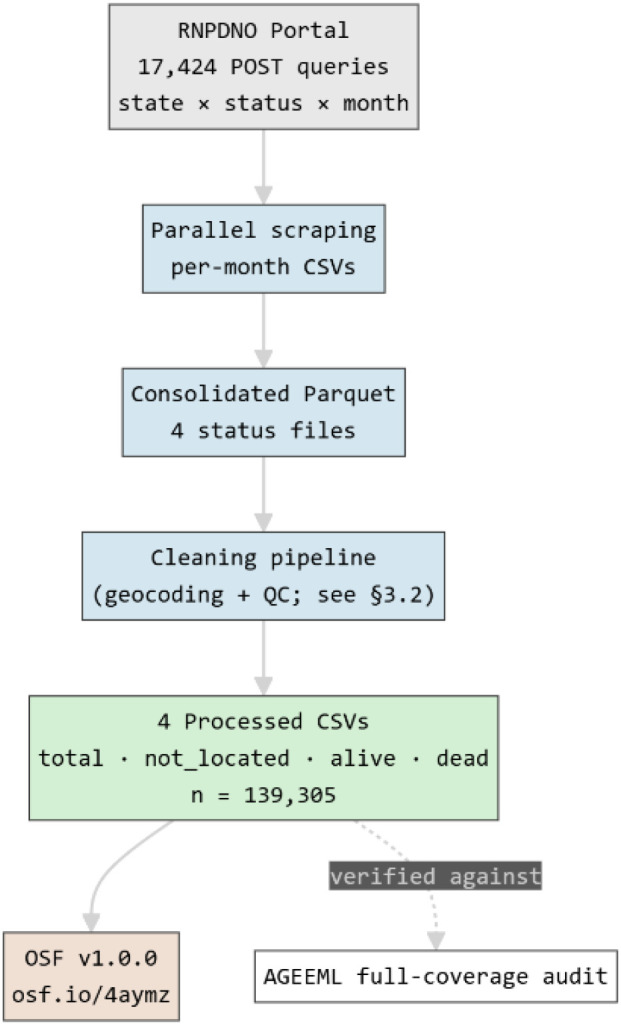
Data acquisition and processing pipeline. Raw records were scraped from the RNPDNO portal via 17,424 state × status × month queries, consolidated into status-level Parquet files, geocoded against AGEEML 2023, and quality-checked. The four processed CSVs (*n* = 139,305 municipality-month observations) were deposited to OSF (v1.0.0) and independently verified against AGEEML for full geographic coverage. Grey: source. Blue: transformation. Green: analytic output. Orange: published artifact. The dashed arrow denotes verification, not data flow.

The dataset was produced by a multi-step pipeline comprising a scraping stage and a cleaning stage. Table 4 summarizes the key files. All code, manual override files, and the Conda environment specification are version-controlled in a public GitHub repository [12], see Fig. 3.

**Table 4:** Key pipeline files and their roles.

Step	File	Description
1	scripts/scrapers/ scrape_rnpdno_single_status.py	Iterates over 33 states and 132 monthly date ranges for one victim-status category. Sends POST requests to the RNPDNO sociodemographic endpoint; parses municipality-level counts from HTML tables embedded in JSON responses. Outputs per-month CSVs, then consolidates into one intermediate Parquet file. Designed for parallel execution (four terminals, one per status).
2	scripts/processing/ normalize.py	Defines two string-normalization functions: normalize_text (standard: lowercase, strip accents, strip punctuation, collapse whitespace) and normalize_text_aggressive (adds abbreviation expansion and removal of the word “de”).
3	scripts/processing/ clean_pipeline.py	Main cleaning pipeline. Per dataset: (a) loads and normalizes raw Parquet; (b) detects discrepancies; (c) applies state corrections; (d) two-pass geographic join against AGEEML; (e) applies manual overrides; (f) assigns sentinel geo-codes; (g) assigns unresolved geo-codes; (h) assembles target schema; (i) aggregates collision duplicates; (j) runs quality checks; (k) writes processed CSV.
4	data/manual/ geo_state_corrections.csv	25 validated corrections where the municipality name unambiguously maps to a different state than reported in the source data. Applied before the geographic join.
5	data/manual/ geo_overrides.csv	36 manually verified mappings of (raw_id_estado, raw_municipio) → cvegeo for cases requiring human disambiguation (e.g., politically renamed municipalities, cross-state boundary ambiguities).
6	data/external/ ageeml_catalog.csv	INEGI AGEEML municipal geo-code catalog (2023 edition) [13]. Reference table for the two-pass geographic join. Encodes 2478 municipalities across 32 states.
7	environment.yml	Conda environment specification for full pipeline reproducibility.

### 4.1 Data extraction

The RNPDNO sociodemographic portal exposes an endpoint at /SocioDemografico/TablaDetalle that accepts POST requests and returns JSON objects containing an Html field with a dynamically rendered HTML table of municipality-level counts. The portal requires session-based authentication with browser cookies; the scraping script loads a pre-obtained cookie from a .env file (the CAPTCHA is solved manually in a browser session; the resulting cookie is then used for automated requests).

The scraper (scrape_rnpdno_single_status.py) processes one victim-status category per invocation (status_id ∈ {0, 2, 3, 7}) and is designed for parallel execution across four terminal sessions. For each (state, year, month) combination, the script sends a POST request specifying the state identifier (id_estado, 1–33), the date range for that month, and the victim-status code. The response contains counts for all municipalities within that state that recorded at least one person in the given month. The script iterates over 33 states (including id_estado = 33, “*se desconoce*”) and 132 monthly periods (January 2015 through December 2025). BeautifulSoup4 parses each HTML table, extracting the municipality name and sex-disaggregated counts (male, female, undefined). Per-month CSV files are written to data/raw/rnpdno/{status_name}/ and subsequently consolidated into one intermediate Parquet file per status in data/raw/rnpdno/consolidated/. A 0.5-second delay is enforced between state-level requests, and a 1.0-second delay between months, to limit load on the portal. Failed requests are retried up to four times with exponential backoff.

### 4.2 Cleaning pipeline

The cleaning pipeline (scripts/processing/clean_pipeline.py) processes each of the four intermediate Parquet files through a deterministic sequence of ten steps: load and normalize, discrepancy detection, state corrections, two-pass geographic join, manual overrides, sentinel assignment, unresolved assignment, schema assembly, duplicate aggregation, and quality checks. The pipeline is written in Polars, with Pandas used for dictionary-based row-level lookups during the geographic join.

### 4.3 String normalization

Municipality names in the source data contain inconsistencies: accent variation, abbreviation, whitespace differences, and politically renamed municipalities. The pipeline normalizes these through two functions defined in normalize.py (Table 5). The aggressive variant expands nine abbreviation patterns (Table 6).

**Table 5:** String normalization functions used in the two-pass geographic join.

Function	Operations	Example
normalize_text()	(1) str + strip; (2) NFD decomposition, drop non-spacing marks (accents); (3) lowercase; (4) replace non-alphanumeric characters with spaces; (5) collapse whitespace.	"San Miguel de Allende" → "san miguel de allende"
normalize_text_aggressive()	All normalize_text() operations, then: (6) expand abbreviations (Dr→doctor, Gral→general, Cd→ciudad, Sta→santa, Sto→santo, etc.); (7) remove standalone word “de”.	"Cd. de Allende" → "ciudad allende"

**Table 6:** Abbreviation expansions applied by normalize_text_aggressive().

Abbreviation	Expansion	Abbreviation	Expansion
Dr	doctor	Sta	santa
Gral	general	Sto	santo
Cd	ciudad	mto	maestro
Prof	profesor	ing	ingeniero
Lic	licenciado		

String normalization can introduce three classes of ambiguity in the geographic join, each handled by a dedicated downstream step. First, homonyms across states (e.g., several states have a "Pueblo Nuevo") are resolved by restricting all lookups to (cve_estado, normalized_name) pairs in the two-pass join. Second, renamed or alternatively-named municipalities (e.g., Tlaquepaque → San Pedro Tlaquepaque; Solidaridad → Playa del Carmen) are resolved by manual overrides documented at row level in data/manual/geo_overrides.csv. Third, state-municipality inconsistencies in the source data (e.g., a Sonora municipality reported under Sinaloa) are resolved by state-level corrections (data/manual/geo_state_corrections.csv) applied before the join; each correction was independently reviewed by two authors against the AGEEML catalog. Each route is deterministic and git-tracked; no fuzzy matching is used at any step.

### 4.4 State-level corrections

For a subset of records, the reported state identifier is inconsistent with the municipality name. When the municipality name unambiguously maps to a single state in the AGEEML catalog, the state identifier is corrected prior to the geographic join. Twenty-five validated corrections are stored in data/manual/geo_state_corrections.csv. This step runs before the geographic join so that the corrected state key is used in AGEEML lookup.

### 4.5 Two-pass geographic join

Each raw record is matched to an AGEEML entry to assign a standardized five-digit geo-code (cvegeo). The join proceeds in two passes:

**Pass 1 – standard join.** Lookup on (cve_estado, normalize_text(municipio)). This resolves the majority of records using exact normalized-name matching within each state.
**Pass 2 – aggressive join (unambiguous only).** For records unmatched in Pass 1, lookup on (cve_estado, normalize_text_aggressive(municipio)), restricted to keys that map to exactly one AGEEML entry within that state. This resolves cases where abbreviations or the word “de” differ between the source data and the catalog.

Records unmatched after both passes proceed to manual overrides, then sentinel assignment, then unresolved assignment.

Empirically, of the 139,495 raw municipality–month–status records ingested, Pass 1 resolved 131,212 (94.06%) and Pass 2 resolved an additional 1136 (0.81%); 6923 records (4.96%) proceeded to manual overrides or sentinel assignment, and 224 (0.16%) carried id_estado = 33 (unknown state). The Pass-2 lookup is by construction free of within-state ambiguity, since the aggressive map is restricted to keys that resolve to exactly one AGEEML entry; the post-hoc audit (audit_output.py) confirms 100% AGEEML conformance across the 139,305 output rows.

### 4.6 Manual overrides

A git-tracked file data/manual/geo_overrides.csv (36 entries) maps (raw_id_estado, normalize_text(raw_municipio)) → cvegeo for cases requiring human disambiguation (e.g., politically renamed municipalities, cross-state boundary ambiguities). Each override is validated against the AGEEML catalog at load time. Overrides are applied only to rows still unmatched after the two-pass join.

### 4.7 Sentinel geo-code assignment

Records that remain unresolvable are assigned sentinel geo-codes according to hardcoded rules in the pipeline: (a) records with id_estado = 33 (“*se desconoce*” in the source) are assigned cvegeo = 99,998, cve_estado = 99; (b) records where the normalized municipality string equals “sin *municipio de referencia*” are assigned cve_mu*n* = 998; (c) records where the normalized municipality string equals “*se desconoce*” are assigned cve_mu*n* = 999. For category (b) and (c) with a known state, the cvegeo is constructed as cve_estado + cve_mun (e.g., 11,998 for Guanajuato with no municipal reference).

### 4.8 Unresolved geo-code assignment

Records that remain unmatched after overrides and sentinel assignment are assigned a dedicated unresolved geo-code (cvegeo = 99,999, cve_estado = 99, state = “unresolved”) rather than being discarded, preserving the full aggregate person-counts. Across the four processed files, 4 rows (4 persons) carry the unresolved code, corresponding to two municipality names that cannot be disambiguated: Pueblo Nuevo under Sinaloa (no such municipality exists in the AGEEML catalog for that state) and Ocampo under Ciudad de México (Ocampo exists in six other states but not in Ciudad de México).

### 4.9 Duplicate aggregation

State corrections can reassign a raw record to a state that already has its own entry for that municipality–month (e.g., a record for Aguascalientes mistakenly reported under Jalisco is corrected to state 01, colliding with the existing Aguascalientes row). The pipeline detects rows that share the same (cvegeo, year, month, status_id) key after all join steps and aggregates them by summing person counts, retaining the first non-null state and municipality name.

### 4.10 Automated quality checks

The pipeline executes the following checks before writing output:

**Schema validation:** all twelve columns present with expected types.
**Duplicate detection:** zero duplicate rows remain on the (cvegeo, year, month, status_id) key after aggregation.
**Sex–total consistency:** total = male + female + undefined verified for every row.
**Temporal coverage:** year range [2015, 2025] and month range [1,12] verified.
**Cross-status consistency:** the aggregate person-count in the status-0 (total) file is compared against the sum of the three sub-status files (status_id ∈ {2, 3, 7}). As of the reference extraction, the total file records 269,507 persons while the sub-status sum yields 269,164 persons—a difference of 343 persons (0.127%), attributed to asynchronous updates in the RNPDNO portal.

All quality metrics are written to logs/quality_report.csv.

### 4.11 Validation

Validation procedures complement the runtime checks above with additional layers of quality assurance, each git-tracked in the public repository.

First, the automated audit script (scripts/processing/audit_output.py) verifies every processed row (*n* = 139,305 across the four CSV files) against the AGEEML 2023 catalog after the pipeline completes. The audit is byte-deterministic across runs and passes on the v1.0.0 release snapshot.

Second, an iterative discrepancies log (data/manual/discrepancies.csv) records 71 name-level and code-level anomalies surfaced during cleaning of the source data, each linked to its resolution route: state correction, manual override, or sentinel assignment.

Third, manual interventions are documented at row level. The 36 entries in data/manual/geo_overrides.csv each carry a citation to the INEGI source that motivated the override; the 25 entries in data/manual/geo_state_corrections.csv are deterministic state-aware reassignments validated against AGEEML uniqueness.

The cross-status residual of 343 persons (0.127%) noted in the runtime checks, attributed to asynchronous portal updates, lies below the 1% threshold enforced by the audit.

### 4.12 Software environment

Table 7 lists the required software packages. Environment reproducibility is managed via the Conda environment file (environment.yml) included in the repository.

**Table 7:** Software dependencies.

Package	Version	Purpose
python	3.10	Core language
polars	≥1.0	Tabular data processing (clean_pipeline.py)
pandas	≥2.0	Scraper consolidation; geo-join lookups
pyarrow	≥14.0	Parquet I/O
requests	≥2.31	HTTP session management (scraper)
beautifulsoup4	≥4.12	HTML table parsing (scraper)
python-dotenv	≥1.0	Environment variable management (browser cookie)
matplotlib	≥3.7	Figure generation
seaborn	≥0.12	Statistical visualization

## Limitations

The RNPDNO portal is updated continuously as cases are registered, reclassified, or resolved; the dataset represents a point-in-time snapshot extracted in March 2026 and does not capture subsequent modifications. The portal's endpoint, HTML structure, and authentication mechanism may change without notice; long-term reproducibility of the extraction pipeline against future portal states is not guaranteed. A subset of 4141 rows (12,210 persons) carry sentinel geo-codes indicating unresolvable geographic information in the source data; these records are retained for aggregate completeness but cannot be assigned to specific municipalities. The cross-status consistency check reveals the total file contains 343 more persons (0.127%) than the sum of the three sub-status files, attributable to asynchronous portal updates during extraction rather than pipeline error. Municipality boundary changes enacted by INEGI after the 2023 AGEEML catalog edition are not reflected. The scraper authenticates via browser cookies obtained after manual CAPTCHA resolution; session expiration during long extraction runs required periodic manual re-authentication. The dataset contains only sex-disaggregated counts; additional dimensions available in the portal (age, nationality, associated crime type, hypothesis of non-location) were not extracted. The open-source pipeline can be adapted to extract these dimensions. Finally, RNPDNO counts represent administrative registrations, not verified events. Underreporting, particularly in regions with limited institutional capacity, is a recognized property of the source system that this dataset inherits. Two administrative events alter the data-generating process within the panel window: a 2023 verification process carried out by the Secretariat of Welfare (*Secretaría de Bienestar*) reclassified part of the registry, and the LGMDFP reform of 16 July 2025 introduced a real-time data-entry mandate (Art. 107) [3]. The structural character of these limitations, including the operational rather than statistical vocation of the registry, has been documented by the Inter-American Commission on Human Rights [5]. The located-dead series functions as a lower bound on lethal outcomes, since matching disappearance reports to unidentified human remains is constrained by the documented backlog within the national forensic system [5]. The dataset follows semantic versioning; each release carries its own DOI on OSF, while the project DOI (10.17605/OSF.IO/4AYMZ) always resolves to the latest version. This release is v1.0.0.

## Ethics Statement

The authors have read and follow the ethical requirements for publication in Data in Brief and confirm that the current work does not involve human subjects, animal experiments, or any data collected from social media platforms. The RNPDNO dataset is derived exclusively from publicly accessible administrative records published by the Mexican federal government through the CNB under the LGMDFP [3], Article 103 of which mandates that the National Register contain a section accessible to the general public. All data are presented in aggregated, municipality-level form; no individual-level identifiers (names, dates of birth, case numbers, or biometric data) are present in any of the processed files. No Institutional Review Board (IRB) approval or ethics committee clearance is required for secondary analysis of these public administrative data.

## CRediT Author Statement

Marco A. Escobar: Conceptualization, Data curation, Formal analysis, Methodology, Software, Writing – original draft. Gerardo Reyes-Guzman: Validation, Writing – review & editing, Supervision. Abraham Sanchez Ruiz: Validation, Writing – review & editing, Supervision.

## Funding

This work was supported by Universidad La Salle Bajío [grant number ITC2025–18].

## Declaration of Generative AI and AI-assisted Technologies in the Writing Process

During the preparation of this work, the authors used Claude (Anthropic) to assist with code generation for data extraction and processing, and with the refinement of manuscript text for clarity and conciseness. After using this tool, the authors reviewed and edited the content as needed and take full responsibility for the content of the publication.

## Declaration of Competing Interest

The authors declare that they have no known competing financial interests or personal relationships that could have appeared to influence the work reported in this paper.

## Data Availability

OSF)A municipality-level, monthly dataset of disappearance cases and outcomes in Mexico from the National Registry of Disappeared Persons, 2015–2025 (Original data).
